# Assessing Changes in Household Socioeconomic Status in Rural South Africa, 2001–2013: A Distributional Analysis Using Household Asset Indicators

**DOI:** 10.1007/s11205-016-1397-z

**Published:** 2016-06-28

**Authors:** Chodziwadziwa W. Kabudula, Brian Houle, Mark A. Collinson, Kathleen Kahn, Stephen Tollman, Samuel Clark

**Affiliations:** 1 MRC/Wits Rural Public Health and Health Transitions Research Unit (Agincourt), School of Public Health, Faculty of Health Sciences, University of the Witwatersrand, Johannesburg, South Africa; 2 Department of Population Health, London School of Hygiene and Tropical Medicine, London, UK; 3 School of Demography, The Australian National University, Canberra, Australia; 4 Institute of Behavioral Science, University of Colorado at Boulder, Boulder, CO USA; 5 INDEPTH Network, Accra, Ghana; 6 Umeå Centre for Global Health Research, Division of Epidemiology and Global Health, Department of Public Health and Clinical Medicine, Umeå University, Umeå, Sweden; 7 Department of Sociology, University of Washington, Seattle, WA USA

**Keywords:** Agincourt, South Africa, Health and Demographic Surveillance System (HDSS), Socioeconomic status (SES), Household assets, Absolute index, Principal component analysis, Multiple correspondence analysis, Relative distribution methods

## Abstract

Understanding the distribution of socioeconomic status (SES) and its temporal dynamics within a population is critical to ensure that policies and interventions adequately and equitably contribute to the well-being and life chances of all individuals. This study assesses the dynamics of SES in a typical rural South African setting over the period 2001–2013 using data on household assets from the Agincourt Health and Demographic Surveillance System. Three SES indices, an absolute index, principal component analysis index and multiple correspondence analysis index, are constructed from the household asset indicators. Relative distribution methods are then applied to the indices to assess changes over time in the distribution of SES with special focus on location and shape shifts. Results show that the proportion of households that own assets associated with greater modern wealth has substantially increased over time. In addition, relative distributions in all three indices show that the median SES index value has shifted up and the distribution has become less polarized and is converging towards the middle. However, the convergence is larger from the upper tail than from the lower tail, which suggests that the improvement in SES has been slower for poorer households. The results also show persistent ethnic differences in SES with households of former Mozambican refugees being at a disadvantage. From a methodological perspective, the study findings demonstrate the comparability of the easy-to-compute absolute index to other SES indices constructed using more advanced statistical techniques in assessing household SES.

## Introduction

An individual’s or group’s position within a hierarchical social structure known as socioeconomic status (SES) influences one’s access to and control over desired resources including knowledge, money, power, prestige, and beneficial social connections which shape one’s well-being and life chances (Link and Phelan 1995; Mueller and Parcel 1981; Link and Phelan 2005; Link et al. 2008; Phelan et al. 2010). Therefore, it is important to understand the distribution of SES and its temporal dynamics within a population to ensure that policies and interventions adequately and equitably contribute to the well-being and life chances of all individuals.

In low- and middle-income settings, one of the widely used measures of SES is a composite index constructed from a list of household asset items (Ataguba et al. 2011; Barros et al. 2010; Gwatkin et al. 2007; Hong and Mishra 2011; Hosseinpoor et al. 2006; Minujin and Delamonica 2004; Nkonki et al. 2011; Uthman 2009; Van de Poel et al. 2008; Ziraba et al. 2009). The index is often called a “wealth index” or “asset index” (Howe et al. 2012) and the household asset items on which it is derived from include durable goods, housing characteristics, sanitation and access to services. Balen et al. (2010), Howe et al. (2009, 2012), Montgomery et al. (2000) and Sahn and Stifel (2003) have outlined the theoretical basis for the preference of the asset index as a measure of SES in low- and middle-income settings over “direct” measures such as income, expenditure, and financial assets (e.g., savings and pensions). Supporting reasons range from reliability to time and cost effectiveness. For example, the information required to construct the asset index is relatively easy and inexpensive to collect. Additionally, in low- and middle-income settings, household assets provide a better proxy for a household’s long-run wealth compared to information on income or expenditures; this is due to seasonal variability in earnings, income from potentially multiple and diverse informal activies, high rates of self-employment, likely recall bias and misreporting.

Booysen et al. (2008), Sahn and Stifel (2003) and Ward (2014) are among others who have demonstrated that data on household asset ownership collected at more than one point in time using a standardized questionnaire can be used to construct an asset index to compare and follow up the changes in the distribution of SES within populations. The Agincourt Health and Demographic Surveillance System (HDSS), which is central to the research programme of the MRC/Wits Rural Public Health and Health Transitions Research Unit has collected data on household asset ownership every 2 years since 2001 using a standardized questionnaire in the Agincourt sub-district in rural northeast South Africa. In this paper, we use these data to construct and compare asset indices and to assess the dynamics of SES in the Agincourt HDSS study population over the period 2001–2013. The focus is on the temporal changes in the ownership of various household asset items and the distribution of SES.

## Materials and Methods

### Data Sources

The analysis in this paper is based on data on asset indicators collected by the HDSS. The Agincourt system has collected detailed longitudinal data on vital events including births, deaths, in- and out-migrations, as well as complementary data covering health, social and economic indicators in a predominantly rural population in northeast South Africa every year since 1992 (Kahn et al. 2007, 2012). Until 2006, the study included 21 villages. The study area was extended to 26 villages in 2007. Another five villages were added between 2010 and 2012 in response to an expanding trials and evaluation portfolio. The population, of approximately 115,000 people in 2014, is largely Shangaan-speaking and almost a third are former Mozambican refugees who arrived in the area in the early to mid-1980s and their descendants.

Collection of data on household asset indicators that include construction materials of the main dwelling, type of toilet facilities, sources of water and energy, ownership of modern assets and livestock only started in 2001 and has been repeated every 2 years. To assess changes in the asset indicators over the period 2001–2013, we use only the data collected from households in the original 21 villages.

### Statistical Analysis

There are three parts to the analysis. The first part summarizes changes in ownership of various household assets in the Agincourt study population from 2001 to 2013. The second part involves constructing three composite indices that can be used as a measure of SES from the household asset items. The three indices namely absolute index, principal components analysis (PCA) index and multiple correspondence analysis (MCA) index are among the most widely utilized indices in the literature. The three indices are used to assess the robustness of our findings. Similar to the approach adopted by Howe et al. (2008), the three indices are compared with each other using scatter plots and the percentage of households classified into the same and different SES quintiles. The agreement of classification of households into SES quintiles between indices is assessed using Kappa statistics. The Kappa statistic, which takes values between 0 (no agreement better than chance) and 1 (perfect agreement) measures agreement in classification between two methods taking into account the agreement that is expected based on chance alone (Howe et al. 2008). Also similar to the approach adopted by Balen et al. (2010), the Spearman’s rank correlation coefficient is utilised for further comparisons of the three indices. The last part of the analysis applies the method of relative distributions developed by Handcock and Morris (1998, 1999) to the asset indices to assess changes in the distribution of SES over time in terms of location and shape. This part of the analysis also takes into account ethnic differences in the distribution of SES as a previous study by Sartorius and colleagues covering the period 2001–2007 showed persistent differentials in SES between the South African and Mozambican populations (Sartorius et al. 2013).

#### Construction of Asset Indices

The absolute index that we construct has been utilized by a number of other researchers that have analyzed data from the Agincourt HDSS (Houle et al. 2013; Gomez-Olive et al. 2014; Houle et al. 2014; Madhavan et al. 2012). To construct this index, first the items of each asset indicator are assigned a weight so that increasing values correspond to items associated with higher SES. For example, for the asset indicator wall material, *5* = *brick; 4* = *cement; 3* = *other modern material; 2* = *mud;* and *1* = *other traditional material*. Thereafter, the value assigned to each item of an asset indicator is normalized by dividing it by the value assigned to the item associated with the highest SES. This results in items of a given asset indicator taking values within the range [0, 1]. The asset indicators are then grouped into five broad asset subcategories (modern assets, livestock, power supply, water and sanitation, and dwelling structure). The normalized values of the asset indicators within each subcategory are then summed to yield a subcategory-specific value. Each subcategory-specific value is further normalized so that it too is in the range [0, 1]. Finally, the five subcategory-specific normalized values are summed to produce an overall household asset index that falls in the range [0, 5].

The PCA index was first recommended by Filmer and Pritchett (2001) and is one of the most widely used asset indices (Gwatkin et al. 2007; McKenzie 2005; Minujin and Delamonica 2004). Construction of this index starts by constructing an $n \times p$ matrix, ${\mathbf{X}}$, representing ownership of *p* asset items collected from *n* households. Thereafter, each element of ${\mathbf{X}}$ is normalized by subtracting from it the column mean and dividing the difference by the column standard deviation to produce another $n \times p$ matrix, ${\mathbf{Y}}$. Next, a $p \times p$ correlation matrix, ${\mathbf{R}}$, is computed from the normalized data matrix, ${\mathbf{Y}}$. This is followed by solving the equation $\left( {{\mathbf{R}} - {\lambda }{\mathbf{I}}} \right){\mathbf{V}} = 0$ for ${{\lambda }}$ and ${\mathbf{V}}$, where ${{\lambda }}$ is a vector of eigenvalues, ${\mathbf{I}}$ is an identity matrix and ${\mathbf{V}}$ is a matrix of eigenvectors associated with the eigenvalues in ${{\lambda }}$. Each eigenvector is then scaled so that its sum of squares equals the total variance. The product of the normalized matrix of assets variables, ${\mathbf{Y}}$, and the matrix of scaled eigenvectors, ${\mathbf{V}}^{\varvec{*}}$ produces a set of uncorrelated linear combinations of the asset variables for each household *j*, known as *principal components*. For each household, the number of principal components equals the number of asset items, and the rank of each component corresponds to the rank of its associated eigenvector. The first component is associated with the most dominant (largest) eigenvalue and explains as much as possible of the variation in the original data. The second component is associated with the second largest eigenvalue and explains as much as possible of the remaining variation in the data, subject to being uncorrelated with the first component. Similarly, each subsequent component explains as much as possible of the remaining variation in the data, while being uncorrelated with the other components. Formally, for household *j*, the PCA index is computed as

$$A_{j} = v_{11}^{*} \left( {\frac{{{\text{x}}_{{j1 - {\bar{\text{x}}}_{1} }} }}{{s_{1} }}} \right) + v_{21}^{*} \left( {\frac{{{\text{x}}_{{j2 - {\bar{\text{x}}}_{2} }} }}{{s_{2} }}} \right) + \ldots + v_{p1}^{*} \left( {\frac{{{\text{x}}_{{jp - {\bar{\text{x}}}_{p} }} }}{{s_{p} }}} \right)$$

where *v*_*i*1_^*^ are the elements of the scaled eigenvector associated with the largest eigenvalue, ${\text{x}}_{ji}$ are the asset ownership values for household *j* and asset $i, i \in \left[ {1, 2 \ldots p} \right]$, and ${\bar{\text{x}}}_{i}$ and *s*_*i*_ are respectively, the mean and standard deviation of the asset ownership values across all households for asset item *i*. In our description of the steps to derive the PCA index we have kept the mathematical details to a minimum. More detailed mathematical descriptions of the steps involved in the PCA technique can be found in Everitt and Hothorn (2011), Rencher (2003).

The procedure used to construct the MCA index is similar to the one used to construct the PCA index but does not assume that the data are continuous and that there is a linear relationship between the observations (Traissac and Martin-Prevel 2012; Booysen et al. 2008; Howe et al. 2012). Because all the asset indicators are discrete or categorical, others have argued that the MCA index is the most appropriate asset-based measure of SES (Booysen et al. 2008; Traissac and Martin-Prevel 2012; Asselin and Anh 2008). In constructing the MCA index we follow the guidelines provided by Booysen et al. (2008) and Asselin and Asselin and Anh (2008). First, the indicators of asset ownership of all households are organized into a matrix ${\mathbf{X}}$ of ones and zeros called the “indicator matrix”. In the indicator matrix, each categorical asset indicator is decomposed into a set of mutually exclusive and exhaustive binary categories that each take only the value 0 or 1 such that every household has a ‘1’ in exactly one of each asset’s set of categories and a ‘0’ in the rest of the asset’s categories. Second, a matrix ${\mathbf{S}}$ is calculated by taking the $\chi^{2}$ metric on row/column profiles of ${\mathbf{X}}$. Greenacre (2007) provides the formula for computing ${\mathbf{S}}$ as

$${\mathbf{S}} = {\mathbf{D}}_{r}^{{ - \frac{1}{2}}} \left( {{\mathbf{P}} - {\mathbf{rc}}^{\text{T}} } \right){\mathbf{D}}_{c}^{{ - \frac{1}{2}}}$$

where ${\mathbf{P}}$ is the matrix formed by dividing each element of the matrix ${\mathbf{X}}$ by the sum of its elements, ${\mathbf{r}}$ is a vector whose elements are the sums of the row elements of the matrix ${\mathbf{P}}$, ${\mathbf{c}}$ is a vector whose elements are the sums of the column elements of the matrix ${\mathbf{P}}$, and ${\mathbf{D}}_{\varvec{r}}$ and ${\mathbf{D}}_{\varvec{c}}$ are diagonal matrices formed from ${\mathbf{r}}$ and ${\mathbf{c}}$ respectively. Finally, singular value decomposition (SVD) is then performed on the matrix ${\mathbf{S}}$ to decompose it into three matrices such that ${\mathbf{S}} = {\mathbf{UD}}_{\alpha } {\mathbf{V}}^{\text{T}}$ (Greenacre 2007). The columns of the matrices ${\mathbf{U}}$ and ${\mathbf{V}}$ referred to as left and right singular vectors are respectively the eigenvectors of the matrices ${\mathbf{SS}}^{\text{T}}$ and ${\mathbf{S}}^{\text{T}} {\mathbf{S}}$ and the columns of the diagonal matrix ${\mathbf{D}}_{\alpha }$ known as singular values are the square roots of the common positive eigenvalues of ${\mathbf{SS}}^{\text{T}}$ and ${\mathbf{S}}^{\text{T}} {\mathbf{S}}$. Like in the PCA approach, in constructing a single asset index, the elements in the first column vector of the matrix ${\mathbf{V}}$ derived by the SVD are then used as weights of the asset categories. Consequently, as provided by Booysen et al. (2008), the MCA index score for household *i* is calculated as

$$MCA_{i} = R_{i1} W_{1} + R_{i2} W_{2} + \cdots + R_{ij} W_{j}$$

where *R*_*ij*_ is the response of household *i* to asset category *j* and $W_{j}$ is the MCA weight of asset category *j*.

The PCA and MCA indices are derived from pooled data from all the available years. This approach ensures that indices explain variation over time as well as across households and are not affected by changes in the contribution of particular assets to household welfare (McKenzie 2005). Pooling of the data is not necessary for the absolute index as the procedure used to generate this index assigns the same weight to the same asset item across time.

#### Assessing Distributional Changes in SES

The method of relative distributions that we apply to the three indices to assess trends in the distribution of SES quantifies differences between the distributions of a set of measurements of an attribute of interest from a population at one time period and another set of measurements of the same attribute from a different population, or from the same population at a later time period. It takes the values of one distribution (the comparison distribution) and expresses them as positions in another distribution (the reference distribution) (Handcock and Morris 1998, 1999). Compared to the standard approach of comparing distributions using summary statistics such as mean, median and variance, which do not consider the entire distributions, the relative distribution analytic approach allows direct comparisons between outcomes across the entire distributions and provides insights that may be missed by the former approach.

Taking 2001 as the baseline year, we obtain the relative distribution for each later time period, *t*, using the density function of the percentile rank, *r*, of asset index value,$y$, in 2001 as

$$g_{t} \left( r \right) = \frac{{f_{t} \left( y \right)}}{{f_{0} \left( y \right)}}, \quad 0 < r \le 1$$

where *f*_0_(*y*) and *f*_*t*_(*y*) are the density functions of the asset index values in 2001 and at a later time period respectively. Basically, the relative distribution, *g*_*t*_(*r*), represents the ratio of the population density at asset index value, *y*, at each later time period, *t*, to the density in 2001. When there are no differences between the comparison and reference distributions, the relative distribution is uniform or “flat” (taking a value of 1 throughout). When there are differences between the distributions, the relative distribution “rises” or “falls” depending on the direction of the difference. For example, if the proportion of households at a later time period, *t*, with asset index values equal to the median asset index value in 2001 is less than 50 %, the relative distribution will have a value below 1 at a point on the vertical axis corresponding to 50 % on the horizontal axis.

Following the approach by Handcock and Morris (1998, 1999), the changes in the relative distribution of the asset index values in 2001 and at later time periods are statistically summarized using the entropy statistic and median relative polarization (MRP) index. The entropy statistic used is based on the Kullback–Leibler divergence, which is a measure of the distance between two distributions and is defined by:

$$D\left( {F:F_{0} } \right) = \mathop \int \limits_{ - \infty }^{\infty } { \log }\left( {\frac{f\left( y \right)}{{f_{0} \left( y \right)}}} \right)dF\left( y \right) = \mathop \int \limits_{0}^{1} { \log }\left( {g\left( r \right)} \right)g\left( r \right)dr$$

where *g*(*r*) is the probability density function of the relative distribution of asset index values in the reference and comparison distributions and *F*_0_ and *F* respectively represent the cumulative distribution functions of the reference and comparison distributions of asset index values. We use the entropy statistic to quantify: (1) overall divergence between the comparison and reference distributions; (2) divergence between the location-adjusted reference distribution and the reference distribution; and (3) divergence between the comparison distribution and the location-adjusted reference distribution. The location adjustment used is median adjustment. This is preferred over mean adjustment because of the well-known drawbacks of the mean when distributions are skewed. As for the MRP index, we use it to quantify the extent to which the shape difference between the distributions of asset index values in 2001 and at later time periods takes the form of relative polarization or rising inequality. It is computed as:

$$MRP_{t} = 4\mathop \int \limits_{0}^{1} \left| {r - \frac{1}{2}} \right| \times g_{t} \left( r \right)dr - 1$$

where *g*_*t*_(*r*) is the relative population density at asset index value, $y$ at each time period, *t* weighted by the absolute difference between the baseline rank of *y* and the median, $\left| {r - \frac{1}{2}} \right|$. Its value varies between −1 and 1, with 0 representing no change in the distribution of asset index values at time period *t* relative to the baseline year, positive values representing more polarization (i.e. increases in the tails of the distribution) and negative values representing less polarization (i.e. convergence towards the center of the distribution). In order to distinguish the contributions from the lower and upper tails of the distribution to the overall polarization, the MRP index is decomposed into lower (LRP) and upper (URP) polarization indices defined respectively as:

$$LRP_{t} = 8\mathop \int \limits_{0}^{{\frac{1}{2}}} \left| {r - \frac{1}{2}} \right| \times g_{t} \left( r \right)dr - 1$$

$$URP_{t} = 8\mathop \int \limits_{{\frac{1}{2}}}^{1} \left| {r - \frac{1}{2}} \right| \times g_{t} \left( r \right)dr - 1$$

These indices also vary between −1 and 1 and have similar interpretations as the MRP index.

The analysis of ethnic differences in the distribution of SES between the South African and Mozambican populations use the distribution of the asset index values of the Mozambican households as the reference distribution and that of the asset index values of the South African households as the comparison distribution.

### Software

We use STATA version 13.1 (Stata Corp., College Station, USA) to construct the asset indices and to perform the descriptive analyses. We also utilize the R statistical package *reldist* to conduct the relative distribution analysis (Handcock and Aldrich 2002).

### Ethics Statement

The Human Research Ethics Committee (Medical) of the University of the Witwatersrand reviewed and approved the Agincourt HDSS (protocol M960720 and M081145). At the start of surveillance in 1992, community consent was secured from civic and traditional leadership and has continuously been reaffirmed for over two decades through frequent meetings. This is facilitated by the Agincourt Unit’s LINC (Learning, Information dissemination and Networking with Community) Office. Three local people working under a coordinator in the LINC office regularly engage with Community Development Forums as well as a Community Advisory Group in the study site. Both are elected committees comprising village members. Community Development Forums, the lowest level of local government, include the Induna who represents the Traditional Council. The LINC office ensures that Forum members understand research objectives and results and are able to raise concerns about the Unit’s research in their communities, and provide feedback of research results at community meetings. The Community Advisory Group ensures information flows between the Unit and the community, voices concerns, assesses the potential impact of the Unit’s research on the community, and maintains ongoing dialogue and consultation. At the individual and household level, informed verbal consent is obtained from the head of the household or an eligible adult in the household at each annual follow-up surveillance visit. Prior to conducting any interview, a local fieldworker who is well-trained and versed in the Agincourt HDSS methods and the process of verbal informed consent explains in the local language to the respondent the purpose, aims and justification of the HDSS as well as information about confidentiality, privacy and the right to refuse to participate or withdraw from the HDSS. The responsible fieldworker documents the consent process by marking out the respondent on the household roster as well as recording the fieldworker details and date on the spaces provided at the top of the household roster. A verbal consenting process is normal practice for HDSS and the processes followed in the Agincourt HDSS have continued to be accepted by the aforementioned ethics committee. Furthermore, additional ethical clearance was obtained from the same ethics committee for the primary study reported in this paper (protocol M120488).

### Data Availability

Detailed documentation of the Agincourt HDSS data and an anonymized database containing data from 10 % of the surveillance households are freely available on the Agincourt HDSS website (www.agincourt.co.za). The specific customized data used in this study are available on request to interested researchers.

## Results

### Temporal Changes in Household Asset Ownership

Table 1 shows the percentage of households owning particular asset items in the 21 villages of the Agincourt HDSS over the period 2001–2013. The results indicate substantial increases over time in the proportions of households that own asset items associated with greater modern wealth. One notable change is the increase in the proportion of households with dwellings constructed with either brick or cement walls from 76 % in 2001 to 98 % in 2013. The prevalence of tiles as roof and floor materials of dwellings also respectively increased from 3 and 0.5 % in 2001 to 15 and 14 % in 2013. In addition, the proportion of households using electricity for lighting and cooking respectively increased from 69 and 4 % in 2001 to 96 and 50 % in 2013. Further noticeable changes are the increases in the proportions of households owning stove, fridge, cellphone and car respectively from 41, 40, 37 and 14 % in 2001 to 85, 86, 98 and 20 % in 2013. On the contrary, proportions of households that own asset items associated with traditional wealth such as animal drawn cart and livestock with the exception of chickens have remained persistently low. The prevalence of animal drawn cart remained nearly unchanged from 3 % in 2001 to 1 % in 2013. Similarly, the proportion of households not owning cows or pigs only marginally changed from 85 % in 2001 to 88 % in 2013 for cows and from 96 % in 2001 to 98 % in 2013 for pigs. In addition, not owning goats slightly increased from 87 % in 2001 to 92 % in 2013.

**Table 1:** Percentage of households owning particular assets items in villages of Agincourt HDSS, South Africa, over the period 2001–2013 and weights assigned to each asset indicator in absolute, PCA and MCA SES indices

	Percentage of households owning asset							Asset weights		
	2001 (n = 10,974)	2003 (n = 11,501)	2005 (n = 11,341)	2007 (n = 11,253)	2009 (n = 12,760)	2011 (n = 11,549)	2013 (n = 11,363)	Absolute index	PCA index	MCA index
Type of dwelling’s wall material										
Brick	1.43	2.06	2.52	3.79	6.05	3.98	4.68	5	0.04	1.66
Cement	75.00	78.99	85.26	88.40	88.11	92.42	93.14	4	0.16	0.34
Wood/other modern	1.22	0.37	0.63	0.73	0.68	0.61	0.55	3	−0.04	−2.41
Mud	21.10	17.80	11.12	6.76	4.69	2.49	1.35	2	−0.19	−3.40
Other traditional	1.25	0.77	0.48	0.32	0.46	0.49	0.27	1	−0.05	−3.62
Type of dwelling’s roof material										
Tiles	3.16	4.45	5.60	7.00	9.49	12.37	14.78	3	0.12	2.66
Corrugated iron	90.67	90.71	91.15	90.95	89.38	86.86	84.68	2	−0.05	−0.12
Thatch/other traditional material	6.17	4.84	3.25	2.04	1.13	0.76	0.55	1	−0.11	−4.15
Floor material										
Tiles	0.46	1.01	1.90	2.67	4.58	9.75	13.57	7	0.10	3.28
Cement	90.06	92.17	93.59	94.81	93.62	88.94	85.68	6	0.02	0.01
Carpet	0.25	0.17	0.16	0.20	0.16	0.06	0.12	5	0.00	0.12
Wood/other modern	0.30	0.13	0.10	0.16	0.11	0.10	0.10	4	−0.01	−2.19
Dirt	5.54	4.50	3.45	1.48	0.77	0.88	0.33	3	−0.13	−4.64
Mat	0.20	0.06	0.05	0.06	0.17	0.13	0.06	2	−0.02	−3.09
Other traditional material	3.19	1.97	0.75	0.60	0.60	0.13	0.14	1	−0.09	−4.98
Number of bedrooms in dwelling										
1	36.51	34.14	28.88	25.26	22.36	20.75	19.70	1	−0.20	−1.96
2	32.60	33.72	35.53	35.60	36.80	34.47	32.61	2	−0.01	−0.12
3	20.62	21.35	22.64	24.53	25.27	26.79	27.47	3	0.12	1.22
4	8.15	8.64	10.18	11.36	12.25	13.85	15.52	4	0.10	1.81
5 or more	2.11	2.15	2.76	3.25	3.32	4.14	4.71	5	0.06	1.99
Separate kitchen	61.29	72.90	73.08	79.20	76.80	66.20	71.64	1	0.11	0.43
No separate kitchen	38.71	27.10	26.92	20.80	23.20	33.80	28.36	0		−1.10
Separate living room	47.52	49.46	51.44	55.26	52.98	58.36	61.06	1	0.19	1.08
No separate living room	52.48	50.54	48.56	44.74	47.02	41.64	38.94	0		−1.26
Location of toilet facility										
Inside the dwelling	0.22	0.15	0.19	0.52	0.57	0.76	2.09	4	0.03	4.01
In the yard	56.35	59.80	65.99	71.23	73.22	78.47	81.35	3	0.26	0.88
Neighbour’s compound	18.08	20.21	17.42	16.15	16.82	14.84	12.76	2	−0.14	−1.57
Bush	25.35	19.84	16.39	12.10	9.39	5.92	3.80	1	−0.21	−2.86
Type of toilet facility										
Modern/flush	0.20	0.15	0.25	0.26	0.28	0.54	2.11	4	0.03	4.33
Ventilated improved pit latrine	0.89	0.48	2.20	4.07	11.14	4.34	9.16	3	0.03	0.97
Traditional latrine/pit	59.29	59.54	63.94	69.19	79.16	75.44	72.44	2	0.23	0.78
No facility	39.61	39.83	33.61	26.48	9.42	19.69	16.29	1	−0.27	−2.33
Source of drinking water										
Tap in the house	0.75	0.50	0.62	0.50	1.41	1.21	0.61	6	0.03	2.31
Tap in the yard	17.99	8.90	16.49	23.43	26.36	30.20	31.75	5	0.14	1.45
Tap on the street	65.35	75.90	80.05	74.19	66.76	65.81	60.77	4	−0.12	−0.42
Water truck	0.31	0.06	0.47	0.66	5.26	2.16	6.26	3	0.03	1.07
Well	10.84	14.38	2.19	0.65	0.05	0.20	0.39	2	−0.04	−1.50
Pond. river. dam. rain and other	4.77	0.25	0.19	0.57	0.16	0.42	0.23	1	−0.01	−1.07
Source of power for lighting										
Electricitry	68.66	76.32	89.35	90.38	93.99	95.51	96.44	4	0.22	0.46
Solar	0.06	0.08	0.04	0.98	0.60	0.12	0.18	3	−0.02	−1.92
Battery	0.08	0.10	0.15	0.10	0.10	0.11	0.24	2	0.00	0.07
Other	31.19	23.49	10.47	8.55	5.31	4.26	3.14	1	−0.22	−3.26
Source of power for cooking										
Electricitry	14.39	19.35	21.83	33.69	41.44	43.85	50.00	5	0.16	1.42
Gas	2.38	1.84	2.14	1.48	0.88	0.81	0.41	4	0.03	1.32
Parafin	6.98	5.36	4.41	1.58	0.44	0.24	0.11	3	−0.04	−1.38
Wood	75.72	73.24	71.51	63.11	57.13	55.02	49.38	2	−0.15	−0.69
Other	0.53	0.21	0.11	0.13	0.11	0.08	0.09	1	−0.02	−2.94
Modern assets										
Stove	41.47	42.53	53.44	66.96	75.27	82.96	85.38	1	0.22	1.03
No stove	58.53	57.47	46.56	33.04	24.73	17.04	14.62	0		−1.85
Fridge	40.52	45.63	57.83	68.09	75.02	81.70	86.22	1	0.26	1.13
No fridge	59.48	54.37	42.17	31.91	24.98	18.30	13.78	0		−2.13
TV	53.47	55.21	59.99	64.43	71.64	80.21	84.21	1	0.22	0.96
No TV	46.53	44.79	40.01	35.57	28.36	19.79	15.79	0		−1.96
Video	5.86	7.27	11.81	39.17	56.07	65.88	66.44	1	0.20	1.68
No video	94.14	92.73	88.19	60.83	43.93	34.12	33.56	0		−0.97
Satellite dish	0.26	0.27	0.51	1.59	5.40	12.44	17.65	1	0.11	3.09
No satellite dish	99.74	99.73	99.49	98.41	94.60	87.56	82.35	0		−0.18
Landline phone	3.24	2.05	1.63	2.29	1.17	1.73	0.87	1	0.04	1.71
No landline phone	96.76	97.95	98.37	97.71	98.83	98.27	99.13	0		−0.03
Cellphone	36.91	52.01	75.73	85.10	92.25	94.87	97.74	1	0.18	0.64
No cellphone	63.09	47.99	24.27	14.90	7.75	5.13	2.26	0		−2.11
Car	14.39	13.43	14.65	15.43	17.88	19.61	19.50	1	0.13	1.96
No car	85.61	86.57	85.35	84.57	82.12	80.39	80.50	0		−0.39
Motorbike	0.66	0.32	0.36	0.66	0.47	0.82	0.51	1	0.02	2.12
No motorbike	99.34	99.68	99.64	99.34	99.53	99.18	99.49	0		−0.01
Bicycle	13.21	10.32	10.04	8.41	9.98	9.96	5.76	1	0.05	0.94
No bicycle	86.79	89.68	89.96	91.59	90.02	90.04	94.24	0		−0.10
Livestock										
Animal drawn cart	3.28	2.40	1.89	1.92	2.12	1.73	1.28	1	0.03	1.23
No animal drawn cart	96.72	97.60	98.11	98.08	97.88	98.27	98.72	0		−0.03
No cows	84.91	87.19	87.67	88.04	88.39	87.42	87.88	0	−0.10	−0.19
1–3 cows	6.05	5.13	4.03	3.33	3.34	3.55	3.10	1	0.04	0.72
4–10 cows	5.81	5.37	5.60	5.48	5.32	5.75	5.58	2	0.07	1.47
More than 10 cows	2.70	1.71	2.00	2.63	2.31	2.75	2.80	3	0.05	1.91
Unknown number of cows	0.53	0.59	0.70	0.52	0.64	0.53	0.64	4	0.03	1.88
No goats	87.48	89.79	90.20	90.70	91.86	91.66	92.24	0	−0.04	−0.05
1–3 goats	7.64	4.38	3.94	3.94	4.04	4.08	3.60	1	0.02	0.28
4–10 goats	4.28	4.81	4.53	4.06	3.36	3.39	3.38	2	0.03	0.61
More than 10 goats	0.48	0.83	1.05	1.13	0.57	0.77	0.56	3	0.01	0.82
Unknown number of goats	0.12	0.18	0.27	0.18	0.16	0.10	0.22	4	0.01	1.13
No chickens	37.58	45.33	60.27	61.01	58.11	52.03	60.62	0	−0.04	−0.19
1–10 chickens	33.34	35.95	29.21	21.35	18.88	20.79	18.63	1	−0.01	−0.21
11–40 chickens	20.24	13.76	7.95	12.40	15.16	21.29	12.85	2	0.05	0.73
More than 40 chickens	1.03	0.37	0.33	1.32	1.14	1.63	0.88	3	0.02	1.08
Unknown number of chickens	7.81	4.60	2.24	3.94	6.70	4.26	7.02	4	0.03	0.66
No pigs	95.74	97.31	97.41	97.73	98.04	97.94	98.29	0	−0.03	−0.02
1–3 pigs	3.73	2.20	2.02	1.55	1.32	1.33	0.96	1	0.02	0.50
4–10 pigs	0.46	0.40	0.43	0.55	0.50	0.59	0.55	2	0.02	1.74
More than 10 pigs	0.05	0.07	0.10	0.12	0.09	0.13	0.15	3	0.01	2.41
Unknown number of pigs	0.02	0.02	0.04	0.06	0.05	0.01	0.04	4	0.01	1.81

### Comparison of Asset Indices

The last three columns of Table 1 present the weights assigned to each asset item in constructing the three asset indices. For the absolute index, the weights are assigned in such a way that increasing values correspond to items associated with higher SES. For the PCA and MCA indices, positive weights are assigned to items expected to be associated with higher SES (e.g. tiles and cement housing floor materials, bricks and cement housing wall materials and tiles and corrugated iron sheets housing roof materials) and negative weights are assigned to items expected to be associated with lower SES (e.g. mud and other traditional housing floor and wall materials, and thatch and other traditional housing roof materials). However, on average the absolute values of the weights in the MCA index are higher than those in the PCA index. In addition, the ranking of the asset items based on the weights in the MCA and PCA indices show marked differences. From the PCA index, the highest weight is assigned to owning a toilet within the yard followed by owning a fridge and the lowest weight is assigned to not owning any toilet facility followed by sources of power for lighting other than electricity, solar or battery. From the MCA index, the highest weight is assigned to owning a toilet inside the dwelling followed by owning a flush toilet and the lowest weights are assigned to owning a house with the floor made of traditional materials such as dirt.

**Fig. 1:**
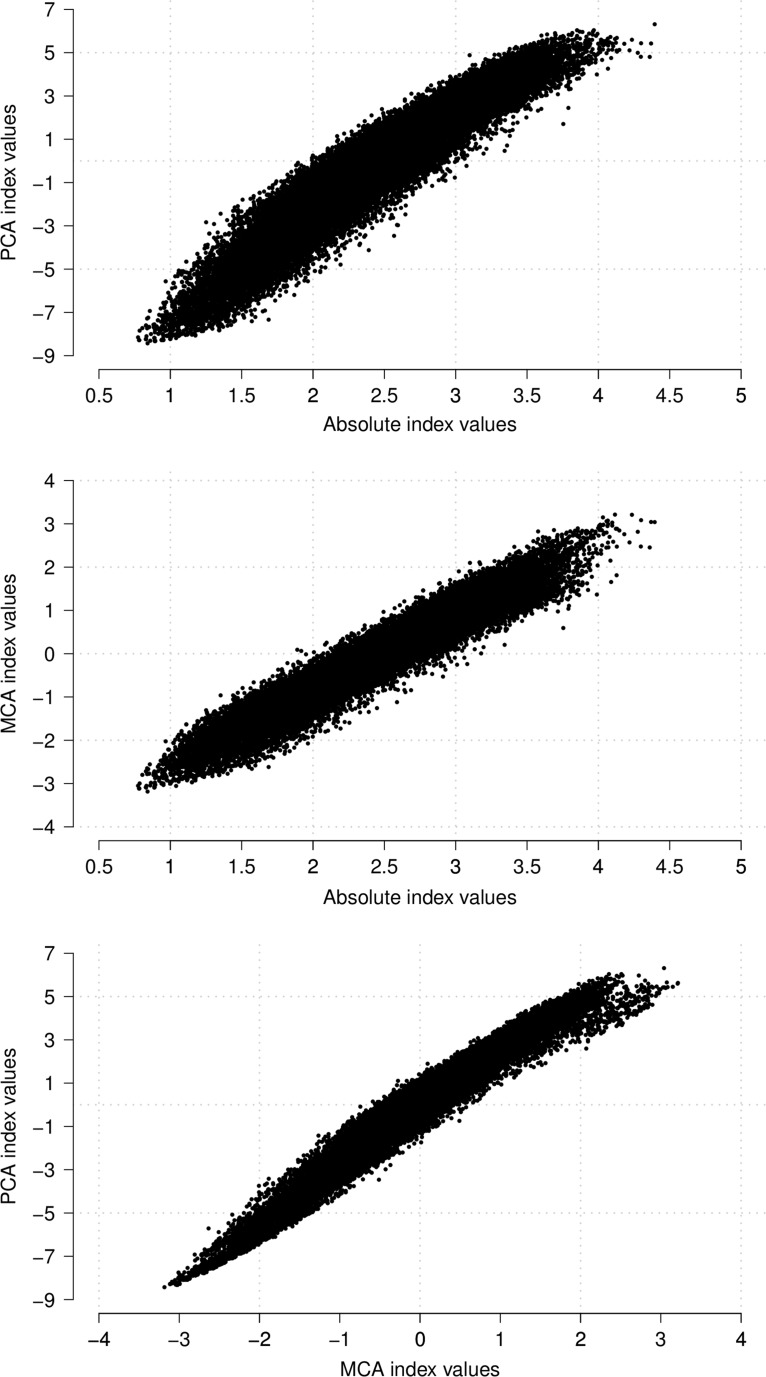
Pairwise comparisons of asset index values

Despite the differences in the weights assigned to the asset items in the three indices, as shown in Fig. 1 and Table 2, the indices are reasonably comparable. Pairwise comparisons between the values of the indices result in correlation coefficients of at least 0.95. In addition, each pair of indices assigns at least 71 % of households in the same SES quintile with Kappa statistics of at least 0.64. Where a pair of indices places households in different quintiles, movement is generally limited to one quintile, with less than 1 % of households moving between two or more quintiles.

**Table 2:** Movement of households between quintiles of absolute, PCA and MCA indices

Indices being compared	Correlation coefficient	Percent of households moving between quintiles			Kappa statistic
		Same quintile	One quintile	Two quintiles	
Absolute and PCA	0.9561	71.28	28.11	0.60	0.6410
Absolute and MCA	0.9668	74.73	25.03	0.24	0.6841
PCA and MCA	0.9835	83.05	16.93	0.02	0.7881

### Distributional Changes in SES

**Fig. 2:**
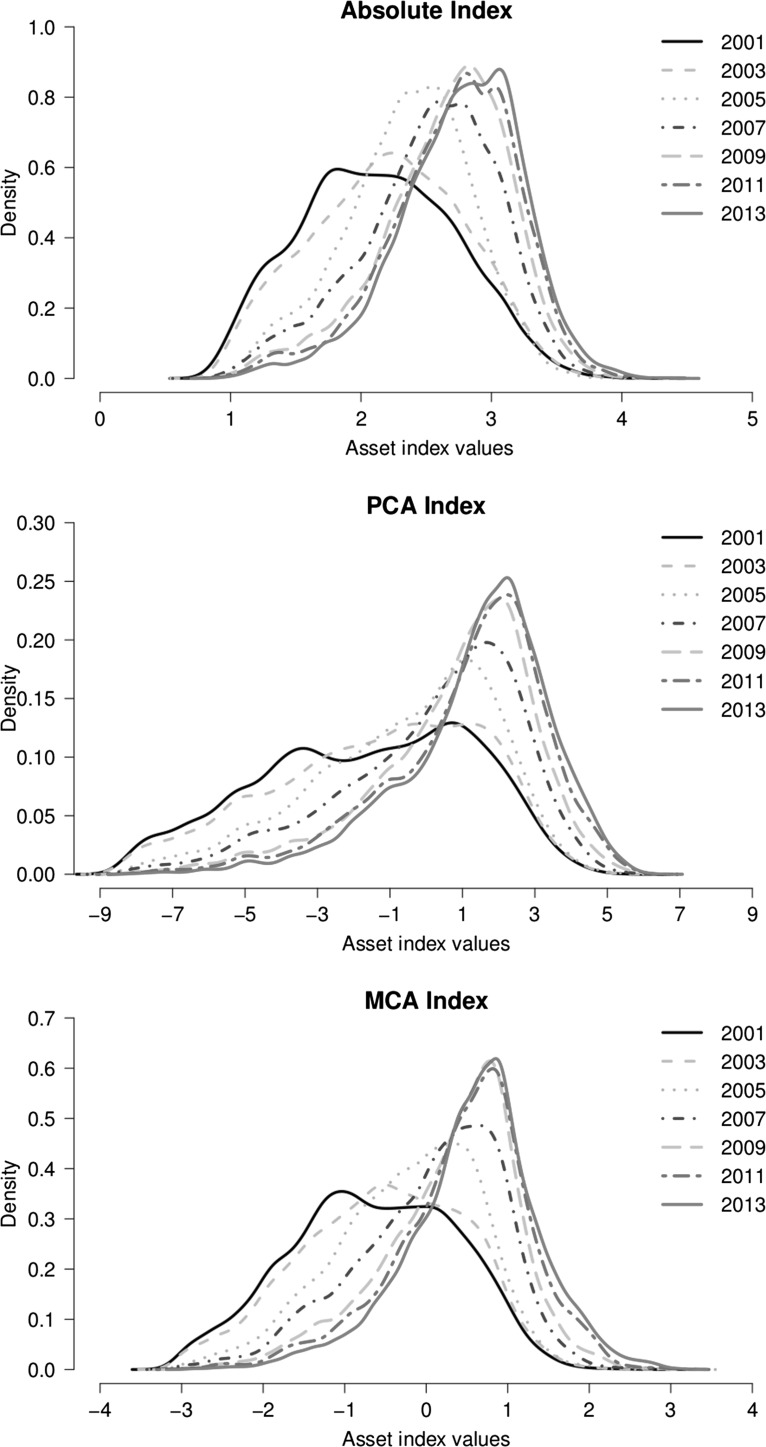
Kernel density estimates of the distribution of SES in the villages of Agincourt HDSS, South Africa, 2001–2013

Figure 2 shows the distribution of SES in the villages of the Agincourt HDSS over the period 2001–2013 based on the absolute, PCA and MCA indices. Overall, from one time period to the next, the mean and median values have persistently shifted to the right across all the three indices. Also it is apparent that the level of variability in the values of all the indices, as depicted by the standard deviation values, has progressively declined over time. Clearly, there has been both location and shape shifts in the SES distribution between 2001 and 2013.

**Fig. 3:**
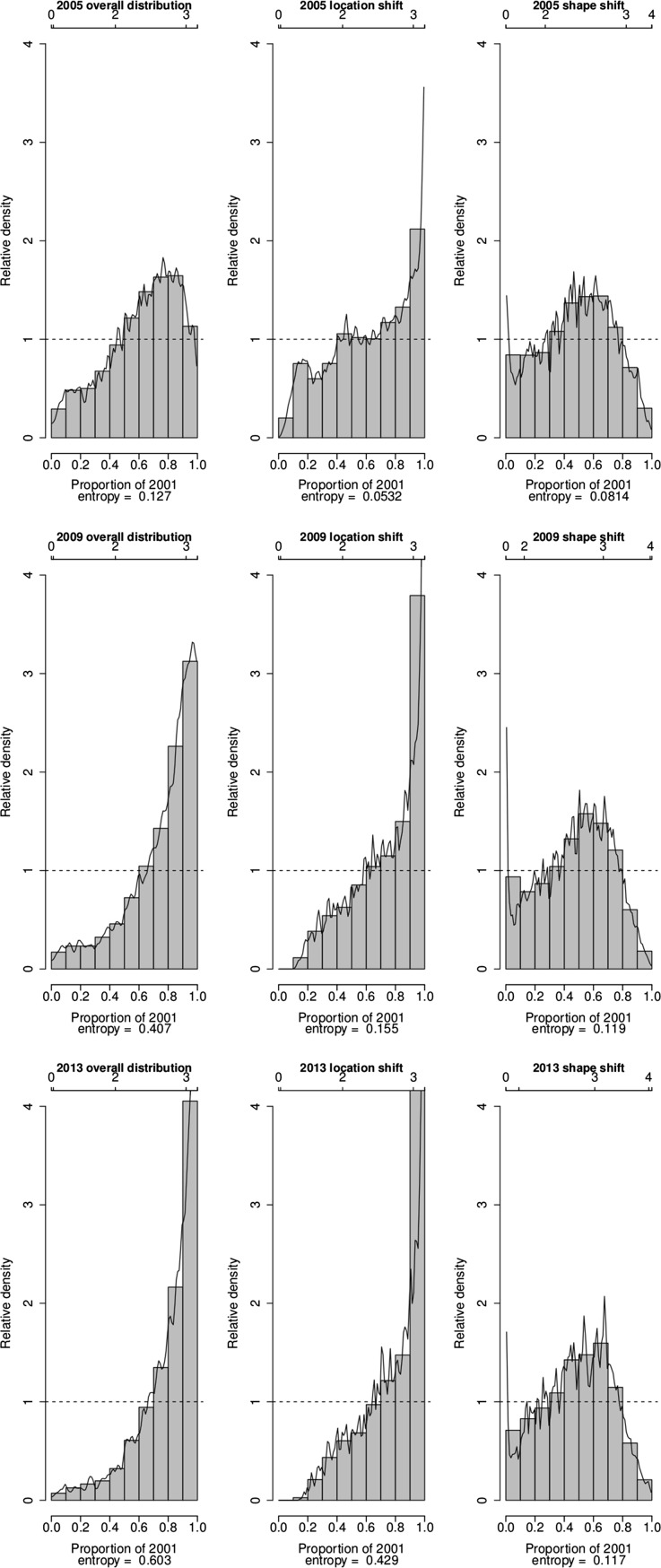
Changes in the relatative distribution of SES in the villages of Agincourt HDSS, South Africa, 2001–2013 based on absolute index

**Fig. 4:**
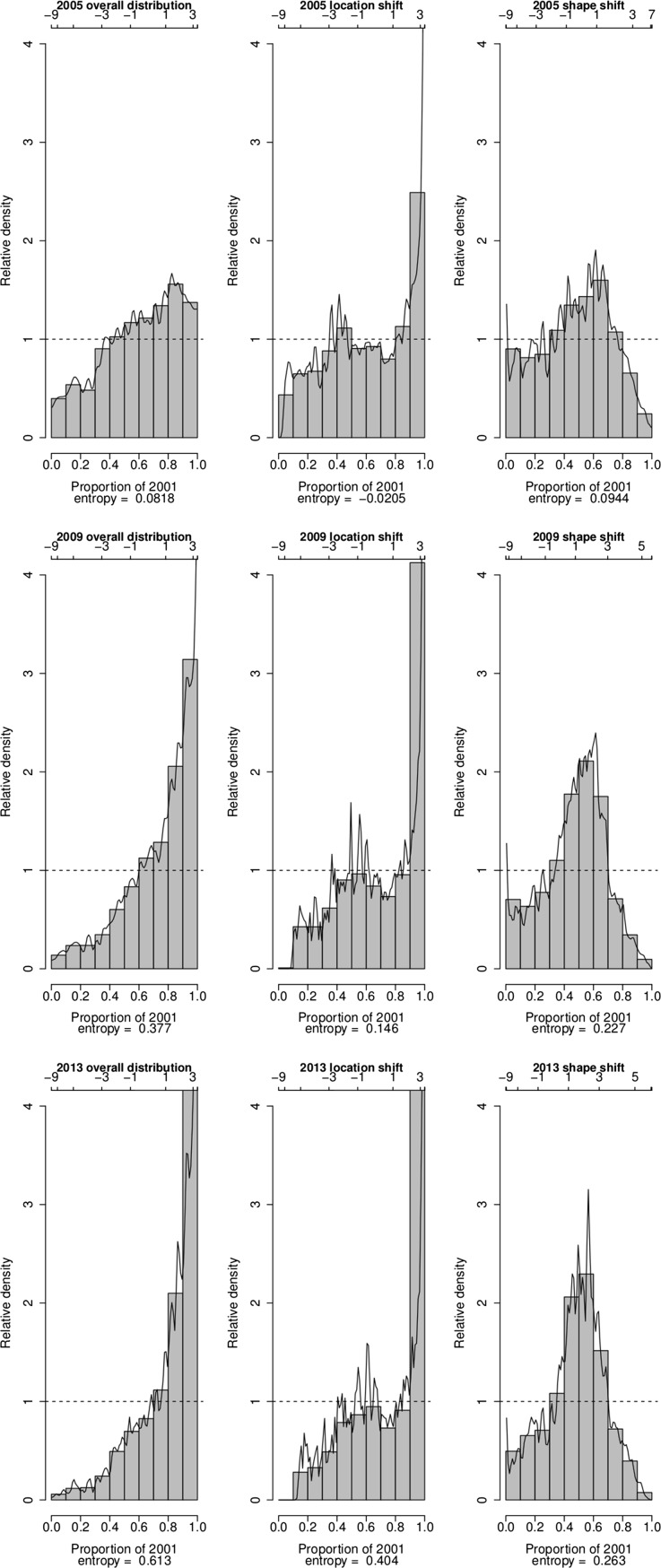
Changes in the relatative distribution of SES in the villages of Agincourt HDSS, South Africa, 2001–2013 based on PCA index

**Fig. 5:**
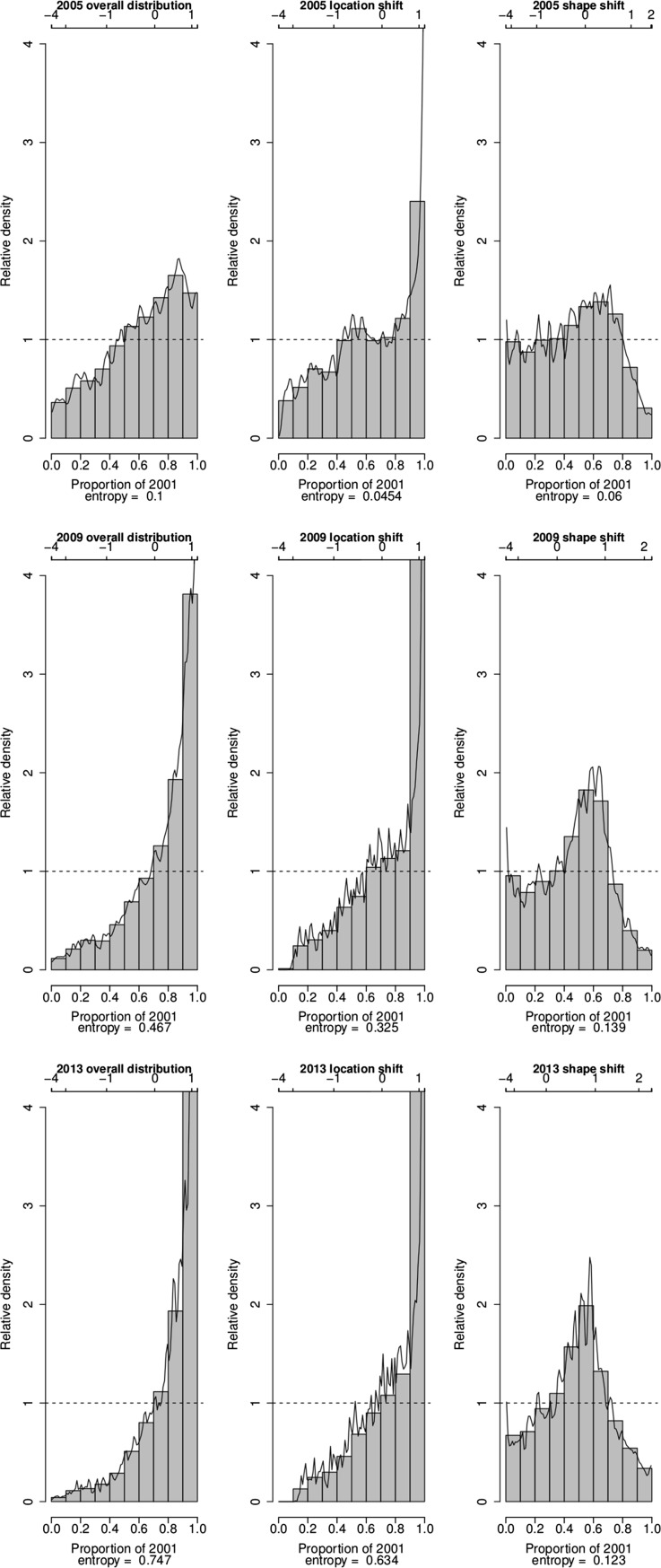
Changes in the relatative distribution of SES in the villages of Agincourt HDSS, South Africa, 2001–2013 based on MCA index

Further insights into the key changes that have occurred in the distribution of the SES of households in the villages of Agincourt HDSS over the period 2001–2013 are provided by plots of the relative distribution of the densities of asset index values in selected years (2005, 2009 and 2013) to the density of asset index values in 2001 in Fig. 3, 4 and 5. The plots of overall distribution show the fraction of households in a particular year that fall into each decile of the 2001 SES distribution. The plots of location shift present the pattern of the relative distribution with no shape but only a location (median) shift in the SES distributions. The plots of the shape shift show the pattern of the relative distribution with no median but only a shape shift in the SES distributions.

In all the three indices, the value of the overall relative distributions is higher than one above the 7th decile of the 2001 distribution from 2009. This means that from 2009 there are higher proportions of households with asset index values that are above the asset index value in the 8th decile of the 2001 distribution. The entropy statistics for the overall relative distribution provide further evidence that irrespective of the index used, over time the distribution of SES has become more divergent from that in 2001. Using the absolute index, the entropy statistics moves from 0.127 in 2005 to 0.407 in 2009 and 0.603 in 2013. Using the PCA index, the entropy statistics moves from 0.0818 in 2005 to 0.377 in 2009 and 0.623 in 2013. Finally, if we use the MCA index, the entropy statistics changes from 0.1 in 2005 to 0.467 in 2009 and 0.747 in 2013.

The relative distributions with location shift illustrate that the effect of the median shift is quite large across all the three indices from 2009. In all the indices, changes in the median alone have caused the proportion of households with asset index values corresponding to the highest decile of the 2001 distribution in 2013 to be more than four times that in 2001. In addition, in all the indices the median shift alone has contributed more than 50 % of the overall entropy between the 2001 and 2013 distributions.

The median-adjusted relative distributions, which expose the effect of changes in distributional shape, show that for all the indices, the proportion of households with asset index values corresponding to the middle deciles (4th to 7th deciles) of the 2001 distribution has been increasing over time. Conversely, the proportion of households with asset index values corresponding to the lower and upper deciles of the 2001 distribution has been decreasing over time. This means that the distribution of SES has consistely become less polarized and is converging towards the middle over the years compared to 2001. Further details on the degree of convergence of the SES distribution from the two tails to the middle are provided by the median, lower and upper polarization indices and their corresponding 95 % confidence intervals, as reported in Table 3. The significantly negative values for the median index confirm that the SES distribution has been converging from the two tails to the middle. The significantly negative values for the lower and upper polarization indices confirm further that the convergence has occurred from both tails of the distribution. However, the large negative values for the upper indices compared to the lower indices indicate that the convergence towards the middle deciles from the upper tail of the distribution has been larger than that from the lower tail.

**Table 3:** Median polarization indices

	Lower CI	Estimate	Upper CI	*p* value
*Absolute index*				
2005 distribution compared to 2001 distribution				
Median	−0.193	−0.178	−0.163	<0.001
Lower	−0.136	−0.106	−0.076	<0.001
Upper	−0.279	−0.250	−0.221	<0.001
2009 distribution compared to 2001distribution				
Median	−0.203	−0.189	−0.174	<0.001
Lower	−0.101	−0.072	−0.043	<0.001
Upper	−0.331	−0.303	−0.274	<0.001
2013 distribution compared to 2001distribution				
Median	−0.224	−0.209	−0.194	<0.001
Lower	−0.159	−0.129	−0.099	<0.001
Upper	−0.318	−0.289	−0.260	<0.001
*PCA index*				
2005 distribution compared to 2001distribution				
Median	−0.201	−0.186	−0.171	<0.001
Lower	−0.127	−0.097	−0.067	<0.001
Upper	−0.304	−0.275	−0.246	<0.001
2009 distribution compared to 2001distribution				
Median	−0.346	−0.332	−0.318	<0.001
Lower	−0.247	−0.218	−0.189	<0.001
Upper	−0.472	−0.446	−0.420	<0.001
2013 distribution compared to 2001distribution				
Median	−0.389	−0.375	−0.361	<0.001
Lower	−0.326	−0.297	−0.269	<0.001
Upper	−0.479	−0.452	−0.426	<0.001
*MCA index*				
2005 distribution compared to 2001distribution				
Median	−0.148	−0.133	−0.118	<0.001
Lower	−0.072	−0.042	−0.011	<0.001
Upper	−0.254	−0.225	−0.195	<0.001
2009 distribution compared to 2001distribution				
Median	−0.242	−0.227	−0.213	<0.001
Lower	−0.114	−0.084	−0.055	<0.001
Upper	−0.398	−0.370	−0.343	<0.001
2013 distribution compared to 2001distribution				
Median	−0.271	−0.256	−0.242	<0.001
Lower	−0.207	−0.177	−0.147	<0.001
Upper	−0.364	−0.336	−0.307	<0.001

**Fig. 6:**
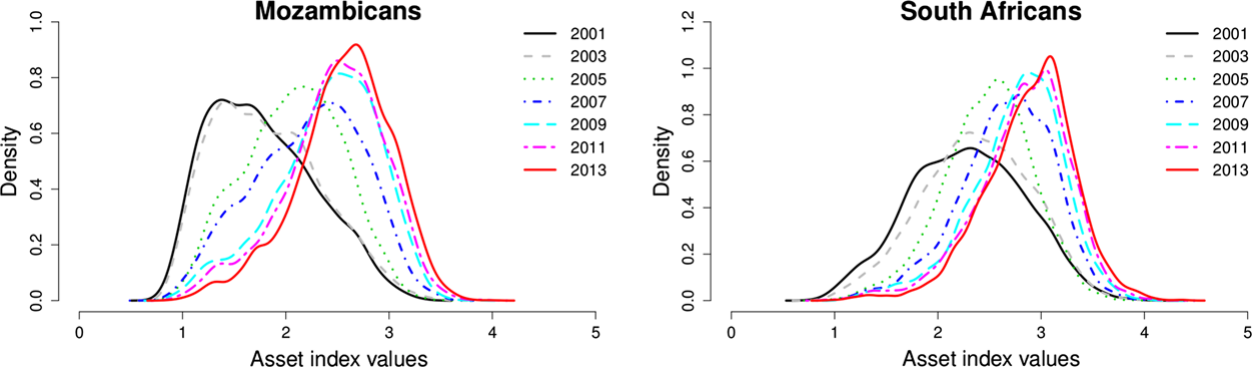
Kernel density estimates of the distribution of SES in the villages of Agincourt HDSS, South Africa, 2001–2013 by ethnicity based on absolute index

**Fig. 7:**
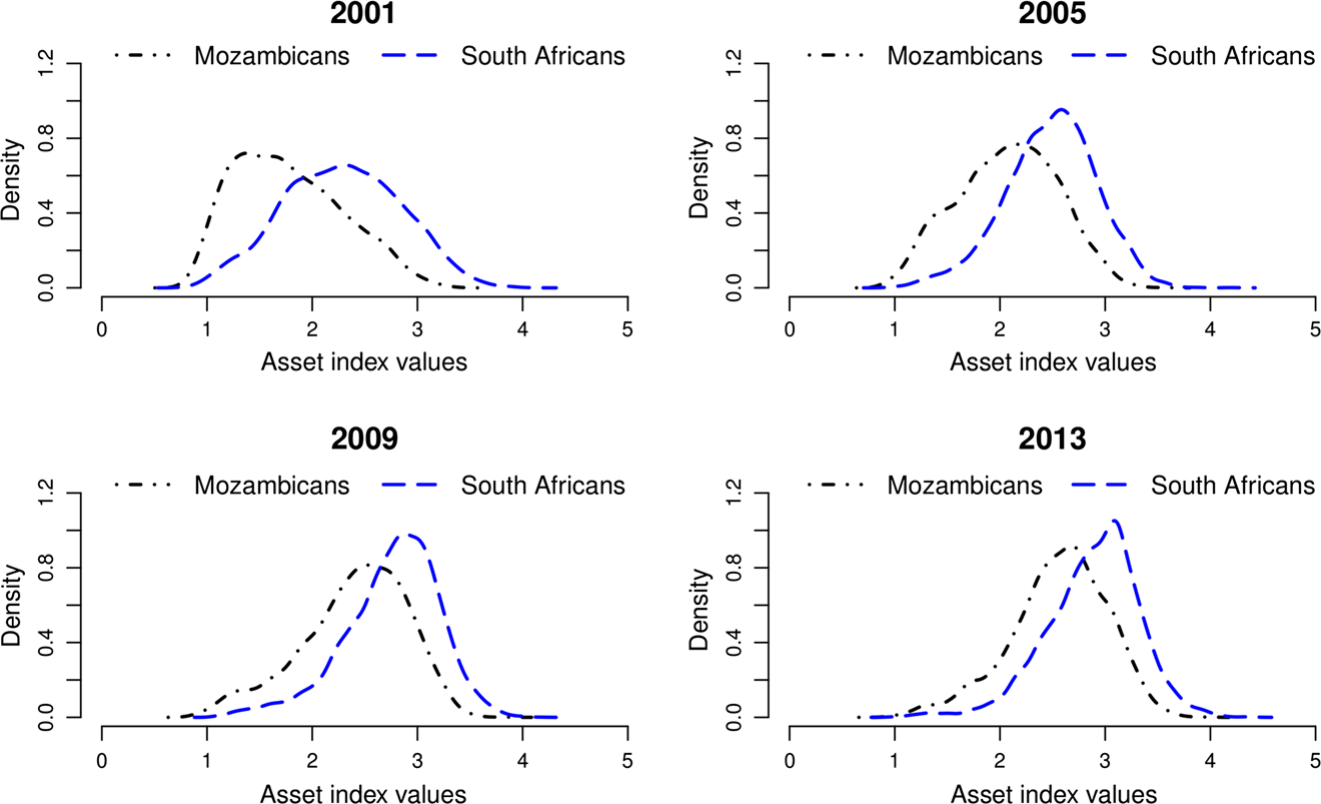
Ethnic differentials in the distribution of SES in the villages of Agincourt HDSS, South Africa, 2001–2013 based on absolute index

**Fig. 8:**
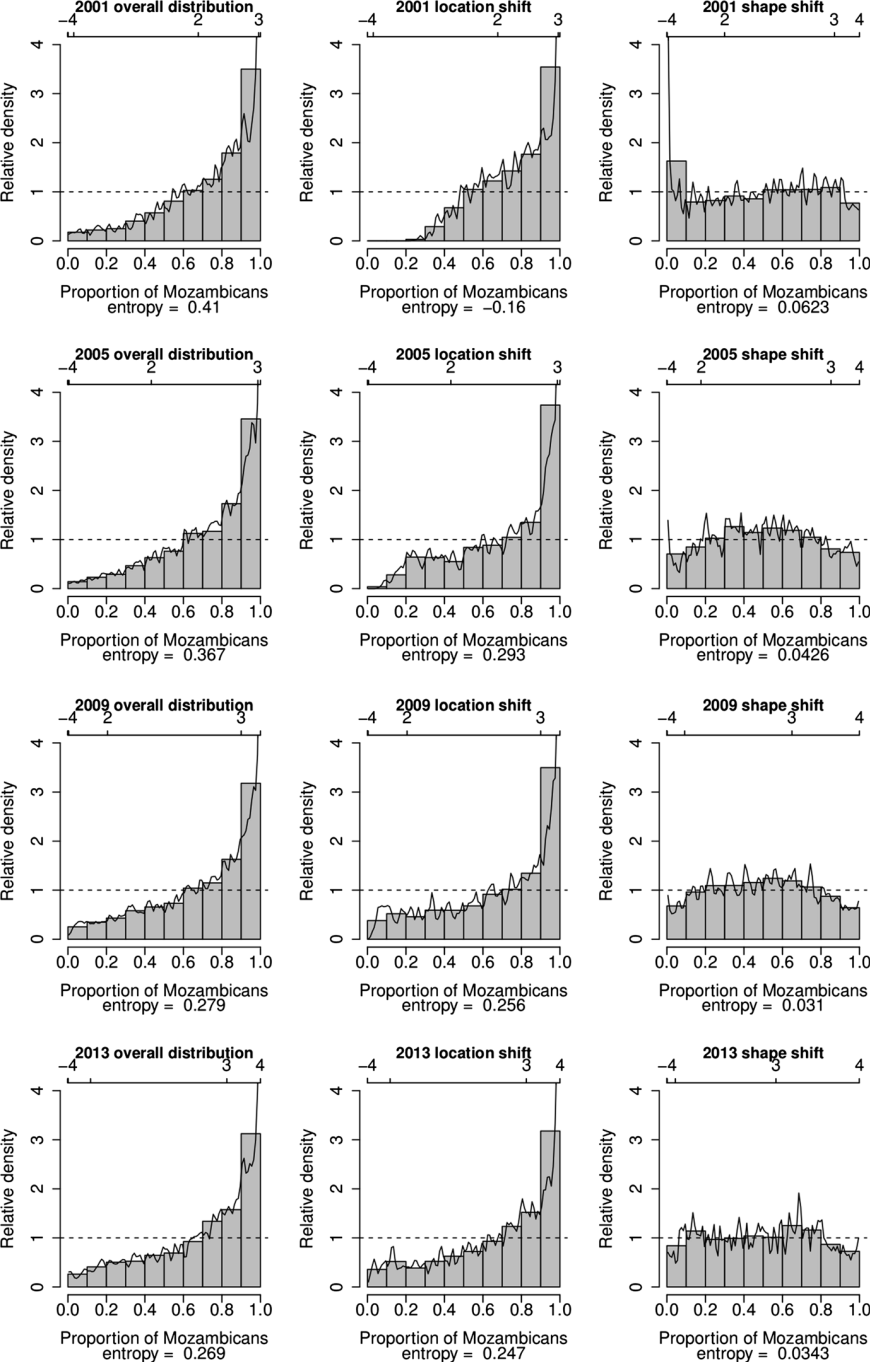
Relative distributions of ethnic differentials in the distribution of SES in the villages of Agincourt HDSS, South Africa, 2001–2013 based on absolute index

The analysis that takes into account ethnic background of the household head shows that improvement in SES has occurred for both South Africans and Mozambicans (Fig. 6). However, at each single point in time the Mozambicans on average have lower SES compared to the South Africans (Fig. 7). A comparison of the distributions of the SES of the two ethnic groups using relative distribution methods indicate that the differences are mainly due to differences in the medians of the distributions (Table 4; Fig. 8). There is little effect of differences in the shape of the distributions.

**Table 4:** Median polarization indices by ethnicity

	Lower CI	Estimate	Upper CI	*p* value
2001				
Median	0.025	0.049	0.073	<0.001
Lower	0.090	0.136	0.182	<0.001
Upper	−0.089	−0.039	0.012	0.066
2005				
Median	−0.134	−0.109	−0.085	<0.001
Lower	−0.148	−0.099	−0.051	<0.001
Upper	−0.168	−0.119	−0.071	<0.001
2009				
Median	−0.125	−0.102	−0.080	<0.001
Lower	−0.125	−0.080	−0.035	<0.001
Upper	−0.169	−0.124	−0.080	<0.001
2013				
Median	−0.072	−0.048	−0.025	<0.001
Lower	−0.073	−0.027	0.020	0.128
Upper	−0.117	−0.070	−0.023	0.002

The analysis is based on the Absolute index

## Discussion

Using pooled data on household assets collected every 2 years from 2001 to 2013 from households of the residents of the Agincourt HDSS, we have assessed the dynamics of SES in a typical South African rural setting. We constructed three asset indices: absolute index, PCA index and MCA index from information on ownership of household assets that include construction materials of the main dwelling, type of toilet facilities, sources of water and energy, ownership of modern assets and livestock. Thereafter, we applied the method of relative distributions to the three indices to assess temporal trends in the distribution of SES.

Our findings indicate that the proportion of households that own assets associated with modern wealth such as stove, fridge, cellphone, car, electricity for lighting and cooking and houses constructed with modern floor, wall and roof materials has substantially increased over time. The increase has persisted beyond the time period covered in an earlier study by Sartorius et al. (2013).

On the contrary, ownership of assets associated with traditional wealth such as livestock has persistently been low. This indicates that unlike other rural populations in sub-Saharan Africa, such as a rural population in Senegal studied by Garenne (2015), traditional wealth contributes to the SES of few households in rural South Africa. This is not surprising since South Africa is a middle-income country. From a policy perspective, the general continuous increase in ownership of assets associated with modern wealth is a positive indicator of the impact of the wide-ranging reforms introduced in South Africa by the post-apartheid government that include the provision of free basic services, such as electricity (50 kWh per household per month), water, sanitation and housing to previously disadvantaged populations the majority of whom live in rural areas (Bhorat and van der Westhuizen 2013). Another important factor has been the implementation of non-contributory social grants provided by the state to vulnerable sectors of the population (Collinson 2010; Lund 2002).

Results from the relative distribution analysis in all the three indices show that the median asset index values have shifted to the right and that the distribution of SES has become less polarized and is converging towards the middle. Worth noting however is that the convergence towards the middle is larger from the upper tail than from the lower tail of the SES distribution. This might be an indication that there has been little or no improvement in the SES of the very poor segment of the population. Further analysis of the charactersitics of the individuals whose SES has persistently remained lower can assist in formulating policies that could bring further improvements in SES. The finding that the SES of the Mozambican households continues to be lower compared to that of South African households suggests that members of the Mozambican households should be among the target of such policies.

From a methodological perspective, the finding that the conclusion drawn from the analysis using the easy-to-compute absolute index are similar to those from the analysis using indices constructed using more advanced statistical techniques such as PCA and MCA demonstrates the utility of the absolute index in assessing people’s SES based on household assets. This finding is consistent with findings by Howe et al. (2008) and Garenne (2015) that SES indices constructed using statistically advanced methods such as PCA offer little advantage over indices constructed using simpler and more intuitive methods such as the absolute index. Since the absolute index has the added property of comparability across time without pooling the data it may be desirable in assessing temporal trends in SES.

Our study uses indices constructed from information on ownership of household assets to assess trends in SES. However, we acknowledge that our approach is by no means the only way to measure SES. Since our indices do not include other factors associated with social exclusion such as gender, education and ethnic background, they may provide only a partial view of the multi-dimensional concept of poverty, inequality and inequity. Nethertheless, our findings provide some interesting insights into the dynamics of SES in rural South Africa in recent years.

## Conclusion

This study has shown that over the period 2001–2013 the rural population in northeast South Africa has experienced significant improvements in ownership of household assets associated with greater modern wealth and polarization of the distribution of SES has declined. However, the movement towards the middle of the SES distribution has been slower for poorer households. Methodologically, the results demonstrate that the absolute index is comparable to other indices constructed using more advanced statistical techniques in assessing people’s SES based on household assets.

## References

[CR1] Asselin LM, Anh VT, Kakwani  N, Silber  J (2008). Multidimensional poverty and multiple correspondence analysis. Quantitative approaches to multidimensional poverty measurement.

[CR2] Ataguba JE, Akazili J, McIntyre D (2011). Socioeconomic-related health inequality in South Africa: Evidence from General Household Surveys. International Journal for Equity in Health.

[CR3] Balen J, McManus DP,  Li YS,  Zhao ZY,  Yuan LP, Utzinge J (2010). Comparison of two approaches for measuring household wealth via an asset-based index in rural and peri-urban settings of Hunan province, China. Emerging Themes in Epidemiology.

[CR4] Barros FC, Victora CG, Scherpbier R, Gwatkin D (2010). Socioeconomic inequities in the health and nutrition of children in low/middle income countries. Revista de Saúde Pública.

[CR5] Bhorat H, van der Westhuizen C (2013). Non-monetary dimensions of well-being in South Africa, 1993–2004: A post-apartheid dividend?. Development Southern Africa.

[CR6] Booysen F, Van Der Berg S, Burger R, Maltitz MV, Rand GD (2008). Using an asset index to assess trends in poverty in seven Sub-Saharan African countries. World Development.

[CR7] Collinson, M. A. (2010). Striving against adversity: The dynamics of migration, health and poverty in rural South Africa. *Global Health Action, 3*, 5080. doi:10.3402/gha.v3i0.5080. 10.3402/gha.v3i0.5080 PMC2882287 20531981

[CR8] Everitt B, Hothorn T (2011). An introduction to applied multivariate analysis with R.

[CR9] Filmer D, Pritchett LH (2001). Estimating wealth effects without expenditure data or tears: An application to educational enrollments in states of India. Demography.

[CR10] Garenne M (2015). Traditional wealth, modern goods, and demographic behavior in rural Senegal. World Development.

[CR11] Gomez-Olive FX, Thorogood M, Bocquier P, Mee P, Kahn K, Berkman L (2014). Social conditions and disability related to the mortality of older people in rural South Africa. International Journal of Epidemiology.

[CR12] Greenacre M (2007). Correspondence analysis in practice.

[CR13] Gwatkin DR, Rutstein S, Johnson K, Suliman E, Wagstaff A, Amouzou A (2007). Socio-economic differences in health, nutrition, and population within developing countries.

[CR14] Handcock MS, Aldrich EM (2002). Applying relative distribution methods in R.

[CR15] Handcock MS, Morris M (1998). Relative distribution methods. Sociological Methodology.

[CR16] Handcock MS, Morris M (1999). Relative distribution methods in the social sciences.

[CR17] Hong R, Mishra V (2011). Effect of wealth inequality on chronic under-nutrition in Cambodian children. Journal of Health, Population and Nutrition.

[CR18] Hosseinpoor AR, Van Doorslaer E, Speybroeck N, Naghavi M, Mohammad K, Majdzadeh R (2006). Decomposing socioeconomic inequality in infant mortality in Iran. International Journal of Epidemiology.

[CR19] Houle B, Clark SJ, Gómez-Olivé FX, Kahn K, Tollman SM (2014). The unfolding counter-transition in rural South Africa: Mortality and cause of death, 1994–2009. PLoS ONE.

[CR20] Houle B, Stein A, Kahn K, Madhavan S, Collinson M, Tollman SM (2013). Household context and child mortality in rural South Africa: the effects of birth spacing, shared mortality, household composition and socio-economic status. International Journal of Epidemiology.

[CR21] Howe LD, Galobardes B, Matijasevich A, Gordon D, Johnston D, Onwujekwe O (2012). Measuring socio-economic position for epidemiological studies in low-and middle-income countries: A methods of measurement in epidemiology paper. International Journal of Epidemiology.

[CR22] Howe LD, Hargreaves JR, Gabrysch S, Huttly SR (2009). Is the wealth index a proxy for consumption expenditure? A systematic review. Journal of Epidemiology and Community Health.

[CR23] Howe LD, Hargreaves JR, Huttly SR (2008). Issues in the construction of wealth indices for the measurement of socio-economic position in low-income countries. Emerging Themes in Epidemiology.

[CR24] Kahn K, Collinson MA, Gómez-Olivé FX, Mokoena O, Twine R, Mee P (2012). Profile: Agincourt Health and Socio-demographic Surveillance System. International Journal of Epidemiology.

[CR25] Kahn K, Tollman SM, Collinson MA, Clark SJ, Twine R, Clark BD (2007). Research into health, population and social transitions in rural South Africa: Data and methods of the Agincourt Health and Demographic Surveillance System. Scandinavian Journal of Public Health.

[CR26] Link BG, Phelan J (1995). Social conditions as fundamental causes of disease. Journal of Health & Social Policy.

[CR27] Link BG, Phelan JC, Mechanic D,  Rogut LB,  Colby DC, Knickman JR (2005). Fundamental sources of health inequalities. Policy Challenges in Modern Health Care.

[CR28] Link BG, Phelan JC, Miech R, Westin EL (2008). The resources that matter: Fundamental social causes of health disparities and the challenge of intelligence. Journal of Health and Social Behavior.

[CR29] Lund F (2002). Social security and the changing labour market: Access for non-standard and informal workers in South Africa. Social Dynamics.

[CR30] Madhavan S, Schatz E, Clark S, Collinson M (2012). Child mobility, maternal status, and household composition in rural South Africa. Demography.

[CR31] McKenzie DJ (2005). Measuring inequality with asset indicators. Journal of Population Economics.

[CR32] Minujin A, Delamonica E (2004). Socio-economic inequalities in mortality and health in the developing world. Demographic Research.

[CR33] Montgomery MR, Gragnolati M, Burke KA, Paredes E (2000). Measuring living standards with proxy variables. Demography.

[CR34] Mueller CW, Parcel TL (1981). Measures of socioeconomic status: Alternatives and recommendations. Child Development.

[CR35] Nkonki LL, Chopra M, Doherty TM, Jackson D, Robberstad B (2011). Explaining household socio-economic related child health inequalities using multiple methods in three diverse settings in South Africa. International Journal for Equity in Health.

[CR36] Phelan JC, Link BG, Tehranifar P (2010). Social conditions as fundamental causes of health inequalities theory, evidence, and policy implications. Journal of Health and Social Behavior.

[CR37] Rencher AC (2003). Methods of multivariate analysis.

[CR38] Sahn DE, Stifel D (2003). Exploring alternative measures of welfare in the absence of expenditure data. Review of Income and Wealth.

[CR39] Sartorius K, Sartorius B, Tollman S, Schatz E, Kirsten J, Collinson M (2013). Rural poverty dynamics and refugee communities in South Africa: A spatial-temporal model. Population, Space and Place.

[CR40] Traissac P, Martin-Prevel Y (2012). Alternatives to principal components analysis to derive asset-based indices to measure socio-economic position in low-and middle-income countries: The case for multiple correspondence analysis. International Journal of Epidemiology.

[CR41] Uthman OA (2009). Decomposing socio-economic inequality in childhood malnutrition in Nigeria. Maternal & Child Nutrition.

[CR42] Van de Poel E, Hosseinpoor AR, Speybroeck N, Van Ourti T, Vega J (2008). Socioeconomic inequality in malnutrition in developing countries. Bulletin of the World Health Organization.

[CR43] Ward P (2014). Measuring the level and inequality of wealth: An application to China. Review of Income and Wealth.

[CR44] Ziraba A, Fotso J, Ochako R (2009). Overweight and obesity in urban Africa: A problem of the rich or the poor?. BMC Public Health.

